# Duplicate MADS genes with split roles

**DOI:** 10.1093/jxb/erw086

**Published:** 2016-03-07

**Authors:** Valérie Hecht

**Affiliations:** School of Biological Sciences, University of Tasmania, Hobart, Australia

**Keywords:** Duplicated B-function genes, functional analyses, MADS-box transcription factors, *Medicago truncatula*, molecular evolution, *PISTILLATA*-like

**Floral morphogenesis is the result of the interaction of an elaborate network of factors, which is relatively well conserved amongst species. In this issue of *Journal of Experimental Botany* (pages 1805–1817), research by Roque *et al.* explains in detail the function of a duplicated pair of genes involved in the formation of petals and stamens in the legume model *Medicago truncatula*.**

Amongst the vast variety in flower shapes and colours in nature, most are composed of the same basic four organs arranged in concentric rings or whorls: sepals, petals, stamens and carpels. Of those, only the two internal organs produce gametes – male gametes in pollen produced in stamens, and female gametes within carpels. Once fertilization occurs, carpel tissues develop into the fruit containing the seeds.

## ABCE model of floral organ specification

Over the past two and a half decades, research on floral development has converged on the now well-known ABCE model for the specification of floral organ identity. This was originally developed in Arabidopsis and snapdragon (*Antirrhinum majus*) based on the analysis of mutants showing homeotic transformations of floral organs.

The initial ABC model postulates that three regulatory gene functions (A, B and C) work in a combinational way to confer organ identity to each whorl, and that A- and C-class genes are mutually antagonistic (Bowman *et al.*, 1991; Coen and Meyerowitz, 1991). Mutants in the A-class genes *APETALA 1* (*AP1*) and *APETALA 2* (*AP2*) have carpels instead of sepals and stamens instead of petals, whereas mutants of the B-class genes *APETALA 3* (*AP3*) and *PISTILLATA* (*PI*) show conversion of petals into sepals and stamens into carpels. Mutants of the C-class gene *AGAMOUS* (*AG*) form petals instead of stamens and sepals instead of carpels. The E-class *SEPALLATA* genes (*SEP1-4*) were added somewhat later to the model, and act together with A, B and C class genes to specify organ identity in all four whorls (Ditta *et al.*, 2004).

This ABCE model explains how floral organs are specified by different combinations of floral organ identity gene activity in the different regions of the flower primordia (Krizek and Fletcher, 2005; Sablowski, 2015). The genes involved in the ABCE model have been isolated in Arabidopsis and all except *AP2* were identified as MADS-box DNA-binding domain transcription factors. Each of them is expressed in a spatially restricted pattern in the floral meristem consistent with the prediction of the initial model (Krizek and Fletcher, 2005; Sablowski, 2015).

MADS-box domain transcription factor genes are involved in controlling most major aspects of a plant’s life, from organ development to cell proliferation and differentiation. MADS-domain proteins act as homo- or heterodimers usually with other MADS-domain proteins, and bind to stretches of conserved DNA sequences also known as CArG-boxes (Kaufmann *et al.*, 2005), but how they achieve their target gene specificity is unclear. The floral quartet model hypothesizes that two dimers of MADS-domain proteins bind to neighbouring CArG-boxes and interact with each other leading to a DNA loop formation resulting in differential regulation of target genes by different quartet complexes (Gramzow and Theissen, 2010; Yan *et al.*, 2016).

This ABCE model has subsequently been generalized to many other flowering plants. In legumes, orthologues of each of the Arabidopsis genes have been identified (Hecht *et al.*, 2005) and several of them have been functionally characterized (Benlloch *et al.*, 2006; Roque *et al.*, 2013; Serwatowska *et al.*, 2014).

## Two *PI-*like genes in *Medicago*

**Fig. 1.**
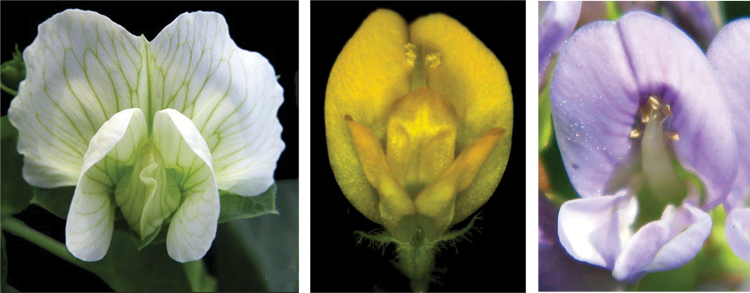
A yellow *Medicago truncatula* flower (centre) beside other flowers of legume species used in the authors’ work. From left to right: garden pea (*Pisum sativum*), *M. truncatula* and alfalfa (*Medicago sativa*). Courtesy of Dr Luis A. Cañas.

Previous work in the authors’ group has characterized organ identity genes in *Medicago truncatula* (Benlloch *et al.*, 2006; Benlloch *et al.*, 2009; Roque *et al.*, 2013; Serwatowska *et al.*, 2014). In the present paper, Roque *et al.* (2016) functionally analyse the contribution of the two *PI*-like genes, *MtPI* and *MtNGL9*, to floral development (Fig. 1).

As might be expected from a *PI* functional orthologue, expression of *MtPI* was detected in floral meristem and in petals and stamens during development. In contrast *MtNGL9* expression was weaker and confined to the epidermal cells of these organs. Both of the corresponding proteins are able to interact with both AP3-like proteins, MtNMH7 and MtTM6, although again, interactions with MtNGL9 seem weaker.

Overexpression of those two genes in Arabidopsis results in homeotic conversion of sepals into petals in various degrees of severity. As expected from the expression analysis, the more severe phenotypes were obtained with *MtPI*. In addition, only *MtPI* was also able to fully complement the Arabidopsis *pi* mutation. Consistent with the major role of *MtPI* in flower development, analysis of the function of both genes in mutants showed no homeotic transformations in *mtngl9* mutants while *mtpi* mutants showed complete conversion of petals into sepals and stamens into carpels.

Phylogenetic analysis showed that the *MtPI* and *MtNGL9* genes arose from a duplication event that occurred prior to speciation of legumes, both genes also being present in *Medicago sativa* (alfalfa), *Pisum sativum* (garden pea), *Lotus japonicus* and *Glycine max* (soybean). Both genes are functional, both expressed and both acting with varying degrees of importance in floral patterning.

Taken together, these results show that *MtPI* is the main regulator in establishing the floral organ fate in whorl 2 and 3, forming petals and stamens in *Medicago*. On the other hand, *MtNGL9* may have a minor role in floral patterning, and the evolution of this gene could lead to possible acquisition of other roles in different developmental processes.

## Other split-role duplicate MADS genes

The authors’ group is the first to systematically examine how MADS-box gene duplications are reflected in gene functions, focusing on pairs of genes in legume floral development. This work, in combination with previous research, analyses the functions of MADS-box domain genes involved in floral patterning in *Medicago*, some of them duplicated and having different roles in flower development (Benlloch *et al.*, 2006; Benlloch *et al.*,2009; Roque *et al.*, 2013; Serwatowska *et al.*, 2014). The two *AP3*-like genes, *MtNMH7* and *MtTM6*, have slightly different B-class functions – *MtNMH7* is more involved in petal identity whereas *MtTM6* has a more important role in stamen identity (Roque *et al.*, 2013). The two *AG*-like genes, *MtAGa* and *MtAGb*, seem each to only have a minor role in C-class patterning of floral organs, both single mutants only showing mild developmental defects (Serwatowska *et al.*, 2014). And of the two *PI*-like genes described here, *MtPI* and *MtNGL9*, only *MtPI* seems to have a role as a B-class gene (Roque *et al.*, 2016).

One of the roles postulated for gene duplication is the generation of new genes and the evolution of biological complexity in an organism (Airoldi and Davies, 2012). Duplicated genes can follow different fates: loss of one duplicate; subfunctionalization, where the function of a single ancestral gene is partitioned between duplicated genes; and neofunctionalization, where a new function is acquired by one of the duplicated genes.

It is interesting to see that different classes of duplicated MADS-box genes have undergone different fates. Syntheny studies have shown that Arabidopsis and *Antirrhinum* have lost one copy of the *AP3/TM6* genes (Causier *et al.*, 2010), while evidence of subfunctionalization of the two *AP3-*like genes appears in legumes, allowing independent control of petal and stamen development (Roque *et al.*, 2013). In the case of the *PI*-like genes, results obtained in *Medicago* probably point to the fact that redundancy has relaxed the functional constraint on one of the two duplicates (*MtNGL9*). This also contrasts with the *AG*-like genes, *AGa* and *AGb*, where single mutants both have similar mild phenotypes.

It would also be interesting to see if *MtNGL9* has a residual function in the absence of B-class function driven by *MtPI* and both *AP3*-like genes. Future studies will no doubt go on to further test the relationships among these genes in multiple mutant combinations to reveal the effects of this additional redundancy and implications for evolution of the legume flower.
